# Evaluating Silymarin Extract as a Potent Antioxidant Supplement in Diazinon-Exposed Rainbow Trout: Oxidative Stress and Biochemical Parameter Analysis

**DOI:** 10.3390/toxics11090737

**Published:** 2023-08-28

**Authors:** Mahdi Banaee, Federica Impellitteri, Cristiana Roberta Multisanti, Antoni Sureda, Francesca Arfuso, Giuseppe Piccione, Caterina Faggio

**Affiliations:** 1 Aquaculture Department, Faculty of Natural Resources and the Environment, Behbahan Khatam Alanbia University of Technology, Behbahan 6361663973, Iran; 2 Department of Veterinary Sciences, University of Messina, Viale Giovanni Palatucci snc, 98168 Messina, Italyfrancesca.arfuso@unime.it (F.A.); 3 Department of Chemical, Biological, Pharmaceutical and Environmental Sciences, University of Messina, 98168 Messina, Italy; cristiana.multisanti@studenti.unime.it; 4 Research Group on Community Nutrition and Oxidative Stress, Health Research Institute of the Balearic Islands (IdISBa), and CIBEROBN Fisiopatología de la Obesidad la Nutrición, University of Balearic Islands, 07122 Palma de Mallorca, Spain

**Keywords:** antioxidant supplement, diazinon, fish, oxidative damage, silymarin

## Abstract

This study aimed to investigate the effects of diazinon on fish, focusing on hepatotoxic biomarkers and the potential protective effects of silymarin supplementation. One hundred eighty rainbow trout were randomly assigned to four groups: control, diazinon exposed (0.1 mg L^−1^), silymarin supplemented (400 mg kg^−1^), and diazinon + silymarin. Blood samples and liver tissue were collected after 7, 14, and 21 days of exposure to analyze biochemical parameters and oxidative biomarkers. Diazinon exposure in fish resulted in liver damage, as indicated by increased antioxidant enzyme activities in the hepatocytes. Silymarin showed the potential to mitigate this damage by reducing oxidative stress and restoring enzyme activities. Nevertheless, diazinon increased creatine phosphokinase activity, which may not be normalized by silymarin. Exposure to diazinon increased glucose, triglyceride, and cholesterol levels, whereas total protein, albumin, and globulin levels were significantly decreased in fish. However, silymarin controlled and maintained these levels within the normal range. Diazinon increased creatinine, urea, uric acid, and ammonia contents. Silymarin could regulate creatinine, urea, and uric acid levels while having limited effectiveness on ammonia excretion. Furthermore, diazinon increased malondialdehyde in hepatocytes, whereas administration of silymarin could restore normal malondialdehyde levels. Overall, silymarin showed potential as a therapeutic treatment for mitigating oxidative damage induced by diazinon in fish, but its effectiveness on creatine phosphokinase, glutathione reductase, and ammonia may be limited.

## 1. Introduction

Diazinon is an insecticide widely used to control pests in agriculture, on livestock farms, and in residential areas. However, its use has led to contamination of surface waters in many parts of the world, including the United States [1,2,3]. Studies have found traces of diazinon in streams, rivers, and lakes due to runoff from agricultural and residential areas [4,5].

In Iran, studies have reported the presence of organophosphate insecticides, particularly diazinon and its derivatives, in surface waters and surrounding environments. The highest levels of diazinon were observed immediately after insecticide spraying, but no traces were detected after a few months. Despite this, diazinon is a persistent pesticide in aquatic environments due to continuous inputs over time [4,5].

Prolonged exposure to low concentrations of diazinon can have negative effects on fish, such as reduced growth and reproductive issues. It can also increase their susceptibility to infectious diseases. Diazinon is toxic to aquatic organisms like fish, insects, and amphibians, which can absorb the chemical through their gills, skin, or digestive systems. Exposure to diazinon can cause various health problems in these organisms, including impaired growth, reproductive issues, and even death [6,7].

In fish hepatocytes (liver cells), diazinon is primarily metabolized through hydrolysis by esterases, resulting in two metabolites: diazoxon and 2-isopropyl-4-methyl-6-hydroxypyrimidine (IMHP). Diazoxon is a more potent acetylcholinesterase inhibitor than diazinon itself and can be toxic to fish if it accumulates to high levels in the body. Both diazoxon and IMHP can undergo further metabolism by cellular enzymes, including cytochrome P450 enzymes, forming various potentially toxic metabolites [8,9].

Exposure to diazinon can disrupt the balance between reactive oxygen species (ROS) and antioxidants in cells. Diazinon can generate ROS through different mechanisms, including inhibition of the mitochondrial respiratory chain, activation of NADPH oxidase enzymes, and disruption of cellular antioxidant defenses. This oxidative stress can damage cellular components, such as DNA, proteins, and lipids [10,11,12].

The level of oxidative stress and cellular damage resulting from diazinon exposure depends on factors like the dose, duration, route of exposure, and individual variations in antioxidant capacity. Minimizing exposure, consuming an antioxidant-rich diet, and enhancing the environmental and physiological conditions of fish are essential steps to mitigate the effects of diazinon and other environmental toxins [12,13]. These strategies can help promote a balance between ROS and antioxidants and protect against oxidative stress.

Research has demonstrated that antioxidant supplements containing vitamins C, E [14], selenium [15], *Arthrospira platensis* [16], and protexin [17] can scavenge free radicals and lessen oxidative stress in fish exposed to various pollutants. Nasirin et al. [18] discovered that quercetin effectively mitigated the toxicity effects of malathion on various aspects, such as the biochemical, immune, and hematological parameters of common carp.

Silymarin is a compound derived from the milk thistle plant and is often used as a dietary supplement for liver-related conditions. It is believed to possess antioxidant, anti-inflammatory, and hepatoprotective properties that help protect liver cells and support their regeneration. The potential antioxidant activity of silymarin has been extensively studied and may aid in reducing oxidative stress in the body [19,20,21]. Silymarin has been investigated in aquaculture as a potential dietary addition for fish. Some studies suggest that including silymarin in fish diets can benefit their health, growth, and flesh quality. For instance, research on Nile tilapia (*Oreochromis niloticus*) [20], turbot (*Scophthalmus maximus* L.) [21], and grass carp (*Ctenopharyngodon idella*) [22] found that silymarin supplementation improved survival rates and growth performance. The researchers hypothesized that silymarin’s antioxidant and anti-inflammatory properties contribute to liver protection and overall well-being [23]. Other studies have also indicated the potential of silymarin in reducing oxidative stress in organs like the liver, brain, and kidneys [24].

Silymarin is a flavonoid complex obtained from milk thistle and is commonly consumed as a dietary supplement with known hepatoprotective effects [25]. It has been investigated for its potential antioxidant properties, which can counteract the harmful effects of diazinon-induced reactive oxygen species (ROS) and safeguard cells against pesticide-related oxidative stress. Several animal studies have shown that silymarin supplementation can mitigate oxidative damage caused by xenobiotics, decrease lipid peroxidation, enhance antioxidant enzyme activity, and improve overall antioxidant status [20,25].

This study aimed to assess the effects of diazinon exposure on rainbow trout and investigate whether silymarin supplementation could alleviate these effects.

## 2. Materials and Methods

### 2.1. Fish

Rainbow trout (*Oncorhynchus mykiss*) with an average weight of 85 ± 15 g were obtained from a private farm located in Kordan town, Karaj, Iran. The fish were then transferred to the aquaculture laboratory of the Fishery and Environmental Department at Tehran University. Upon arrival, the fish were randomly distributed into 12 closed water recirculating systems, each with a capacity of 1000 L. They were kept in these systems for a minimum of 2 weeks to acclimate to the laboratory conditions, which included temperature—15 ± 2 °C, pH—7.4 ± 0.2, light cycle—16 h light and 8 h dark, and a daily water exchange rate of 80%. The fish were fed commercial pellets formulated specifically for rainbow trout during the acclimation period. The feed was provided at a rate of 2% of their body weight twice daily.

### 2.2. Chemicals

Commercial diazinon (Emulsion, EC 60%) was bought from Partonar Co., Tehran, Iran. Biochemical reagents and oxidative biomarker kits were obtained RANDOX Co., (Crumlin, County Antrim, UK) and ParsAzumon Company (Karaj, Iran). Other chemical materials were acquired from Merck Company (Rahway, NJ, USA).

### 2.3. Diet Preparation

Milk thistle seeds obtained from the Pharmaceutical Co. farm (Goldaru, Isfahan, Iran) were subjected to extraction using chloroform–methanol (2:1, *v*/*v*) in a blender at an elevated temperature. The resulting extract was dried using anhydrous sodium sulfate, and the solvent was removed from the filtrate under reduced pressure at 45 °C. Subsequently, silymarin extracts at concentrations of 400 mg kg^−1^ (*w*/*w*) were mixed with powdered food. The primary active components of the extract included various flavonolignans such as silybin, isosilybin, silydianin, silychristin, dehydrosilybin, dehydrosilychristin, neosilyhermin, silyhermin, and silybinome [26,27]. Each supplemented diet was homogenized with distilled water (1 mL g^−1^) until a uniform mixture was obtained. This mixture was passed through a meat grinder, resulting in extruded string shapes that were then dried in an oven at 55 °C for 12 h. Afterwards, the dried strings were broken to form approximately 10 mm long pellets. These pellets were packed and stored at −20 °C in a freezer until needed. The control diet was prepared using the same process without adding any supplements.

### 2.4. Experimental Design

The fish were divided into four groups through a random assignment process. Group I served as the control group and received a normal diet for 21 days. In Group II, the fish were exposed to a concentration of 0.1 mg L^−1^ diazinon. For Group III, the specimens were fed an enriched diet containing 400 mg of silymarin per 1 kg of feed for 21 days. In Group IV, the fish were exposed to 0.1 mg L^−1^ diazinon while being fed an enriched diet with 400 mg silymarin per 1 kg of feed for 21 days. The waste and leftover feed were gathered and then removed from the tanks by siphoning from the bottom of the tanks. Afterwards, 80% of the water was substituted, and in order to sustain the nominal pesticide concentration in the water, fresh diazinon stock was introduced based on the water replacement rate. Sampling was performed on the 7th, 14th, and 21st days. The concentrations of diazinon [2] and silymarin [19,26] were selected based on previous studies. The study protocol received approval from the Institutional Ethics Committee of the Natural Resources Faculty at Tehran University (IR.TU.NRF. 1389.07.28).

The blood sampling procedure was performed using a syringe equipped with a heparinized collection tube. Anesthesia was administered if necessary. The sampling area around the caudal vein was cleaned with a mild antiseptic solution to minimize the risk of contamination. The needle was carefully inserted into the selected vessel, and the desired volume of blood was withdrawn into the collection tube. Then, the collected blood sample was transferred into appropriate containers, labeled accurately, and handled in accordance with the specific requirements of the analysis. Next, centrifugation was carried out within the range of 6000× *g* for approximately 10–15 min at 4 °C. After centrifugation, the plasma was then transferred to sterile tubes or vials using a pipette or a similar instrument, ensuring caution was exercised to avoid disturbing the underlying layer of red blood cells or any formed clots. The collected plasma should be appropriately labeled with relevant information, date, and other necessary details. To maintain its stability until further analysis or utilization, the plasma is stored at −80 °C.

The clove extract solution was used to numb the fish, and they were subsequently euthanized [16]. Next, the fish was positioned ventral side up and secured in place using pins or hooks if needed. A longitudinal incision along the ventral midline of the fish, from the posterior end towards the head, was made using dissecting scissors or a scalpel. The body cavity was gently spread open by carefully pulling back the flaps of the incision. The liver was carefully lifted and separated from surrounding tissues using forceps or fingers. Once isolated, the liver was detached from any connecting tissues or blood vessels using scissors or a scalpel. Finally, the liver was carefully lifted away from the body cavity.

The tissue sample was transferred into the homogenization container containing the phosphate buffer solution (pH: 7.4). Then, the tissue was then broken down and a uniform mixture was created using the tissue homogenizer. Once the homogenization process was complete, the homogenate was transferred to a suitable container for preparing tissue extract. Finally, the samples underwent centrifugation for 15 min at a speed of 15,000× *g* and at a temperature of 4 °C in a refrigerated centrifuge. The resulting supernatants, obtained after centrifugation, were promptly utilized for measuring oxidative biomarkers using spectrophotometric assays.

### 2.5. Blood Biochemical Parameters

Plasma glucose levels, total protein, albumin, globulin, urea, uric acid, ammonia, and creatinine were assessed using standard methods commonly employed in clinical biochemistry labs. The biochemical parameters were measured using biochemical reagents kits, and reagents were purchased from Pars-Azumon (Alborz province, Karaj, Baharestan industrial town, Iran) [28,29,30].

The activity of creatine kinase (CK) was determined by its interaction with creatine phosphate and ADP, which led to the production of ATP. This reaction was linked to the hexokinase/GDP reaction, resulting in the generation of NADPH. Lactate dehydrogenase (LDH) activity was evaluated by monitoring the conversion of pyruvate to L-lactate through the oxidation of NADH. Aspartate aminotransferase (AST) activity was measured in conjunction with malate dehydrogenase in the presence of NADH. In the alanine aminotransferase (ALT) assay, the enzyme reacted with alanine and α-ketoglutarate to produce glutamate and pyruvate. The measurement of GGT (gamma-glutamyltransferase) activity was carried out using the substrate L-ɣ-glutamyl-3-carboxy-4 nitroanilide and the co-substrate glycylglycine at 405 nm. LDH then converted pyruvate to lactate and NAD+. The changes in absorbance at 340 nm were monitored to quantify these activities. Alkaline phosphatase (ALP) activity was determined based on the enzymatic conversion of p-nitrophenol phosphate to nitrophenol in an alkaline buffer, and the measurement was taken at 405 nm. In order to evaluate the activity of plasma butrylcholinesterase (BChE), a suitable volume of the sample was combined with a cuvette containing a solution composed of substrates including 0.1 M phosphate (pH 8.0), butrylcholine iodide (0.015 M), and dithiobis nitrobenzoic acid (0.01 M). BChE activity was recorded for 180 s at 405 nm [28,29,30].

All these biochemical parameters were measured using a UV/VIS spectrophotometer (UNICO model 2100; Suite E Dayton, NJ 08810, USA) in the laboratory of the Hygienic Department at the University of Tehran.

### 2.6. Oxidative Biomarkers

The total antioxidant capacity of liver homogenates was determined using the ferric-reducing ability of plasma (FRAP) method. This involved the reduction of the ferric tripyridyl-s-triazine (Fe^3+^-TPTZ) complex to ferrous tripyridyl-s-triazine (Fe^2+^-TPTZ) at pH 3.6 and a temperature of 25 °C. The resulting Fe^2+^-TPTZ complex has a strong blue color, which was measured for up to 5 min at 593 nm using a UV/VIS spectrophotometer. FeSO_4_·7H_2_O calibration curves were used for calculations [30,31].

The activities of glutathione reductase (GR), glutathione peroxidase (GPx), and superoxide dismutase (SOD) were assessed using diagnostic reagent kits from RANDOX Co. (Crumlin, County Antrim, UK). GR activity was determined by measuring the reduction of oxidized glutathione (GSSG) in the presence of NADPH, which is oxidized to NADP^+^. Absorbance at 340 nm was monitored to measure the decrease in NADPH. GPx activity was determined by using cumene hydroperoxide as a substrate. In the presence of glutathione reductase and NADPH, the oxidized glutathione (GSSG) was rapidly converted to its reduced form (GSH), with concomitant oxidation of NADPH to NADP^+^. SOD activity was measured at 505 nm using a xanthine and xanthine oxidase system to generate superoxide radicals, which reacted with 2-(4-iodophenyl)-3-(4-nitrophenol)-5-phenyltetrazolium chloride (INT) to produce a red formazan dye. The degree of inhibition of this reaction indicated SOD activity. One unit of SOD was defined as causing a 50% inhibition of the reduction rate of INT under test conditions [30,31].

Catalase (CAT) activity was determined based on the decrease in absorbance at 240 nm. The reaction mixture contained 50 mM K-phosphate buffer (pH 6.5) and 50 mM H_2_O_2_ (Merck), which was diluted in 80 mM K-phosphate buffer (pH 6.5). The activity was calculated using the extinction coefficient for H_2_O_2_. Enzyme activities were expressed per milligram of protein in liver tissue, determined by the Biuret method with bovine serum albumin as a standard [3,32].

### 2.7. Statistical Analysis

The normal distribution of the data was verified using the Kolmogorov–Smirnov test. Statistical analysis was carried out using two-way ANOVA and, subsequently, Duncan’s multiple range test to determine significant differences between multiple treatment groups. The significance level was determined to be *p* < 0.01. The statistical analysis was performed using Graph Pad Prism software (version 8). Data are presented as mean ± standard deviation.

## 3. Results

Fish exposed to diazinon, silymarin, or a combination of both did not experience any fatalities. The study found that fish exposed to diazinon experienced a significant (*p* < 0.01) decrease in the levels of total protein, albumin, and globulins. However, when silymarin was administered to these exposed fish, total protein, albumin, and globulin levels were restored (Table 1). There was no notable distinction observed in the total protein, albumin, and globulins levels between the group of fish that received silymarin supplementation and the control group (Table 1).

The findings indicated that when fish were exposed to diazinon, their glucose, triglyceride, and cholesterol levels increased. However, the presence of silymarin was able to control and maintain these levels within the fish’s plasma to the normal range (Table 1). There was no notable difference observed in the glucose, triglyceride, and cholesterol levels between the group of fish that received silymarin supplementation and the control group on the 7th and 14th days. Nonetheless, glucose and cholesterol were significantly (*p* < 0.01) decreased in fish fed silymarin on the 21st day of the trial (Table 1).

Creatinine, urea, uric acid, and ammonia levels were significantly (*p* < 0.01) increased in fish exposed to diazinon on the 7th, 14th and 21st days of the experiment. Administration of silymarin could restore creatinine and urea contents to normal range in fish exposed to diazinon. Although silymarin could adjust uric acid in the plasma of fish exposed to diazinon on the 7th and 14th days of trial, silymarin could not restore uric acid in the plasma of fish exposed to diazinon on the 21st day of the experiment. Despite the administration of silymarin, ammonia concentration was significantly higher in fish exposed to diazinon than in the control group (Table 2).

**Figure 1:**
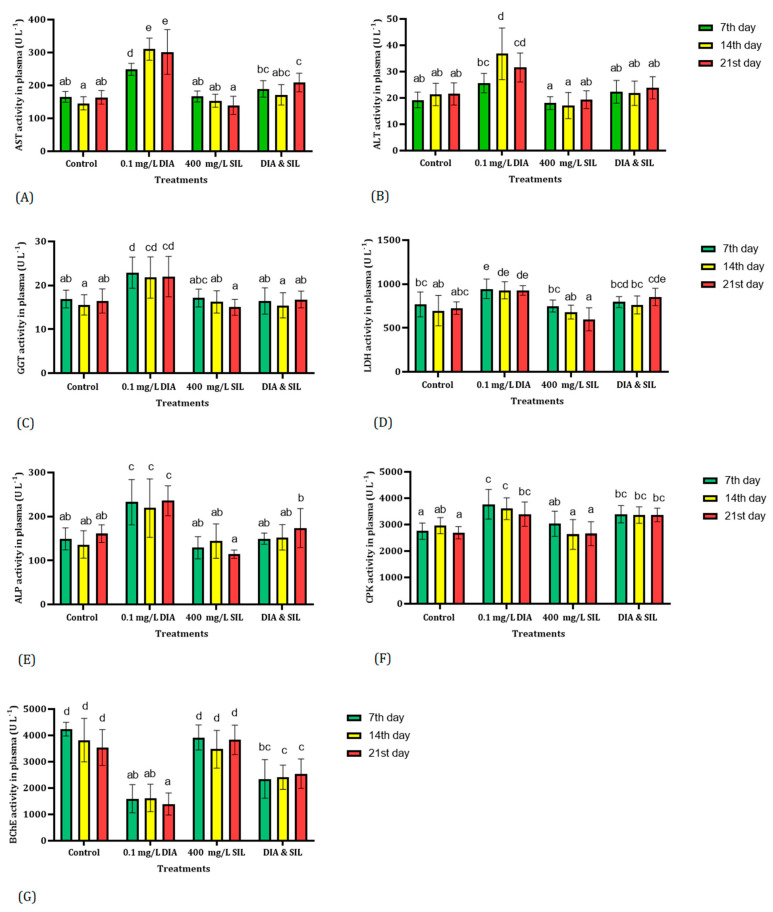
The effect of diazinon, silymarin, diazinon + silymarin on plasma enzyme activities. Values with distinct letters in a column indicate a significant difference (*p* < 0.01). Aspartate amino transferase (**A**), alanine amino transferase (**B**), gamma-glutamyltransferase (**C**), lactate dehydrogenase (**D**), alkaline phosphokinase (**E**), creatine phosphokinase (**F**), and butrylcholinesterase (**G**).

Changes in the activity of enzymes in the plasma of fish are illustrated in Figure 1. The findings indicated that silymarin did not have a significant impact on the activities of AST, ALT, GGT, LDH, ALP, CPK, and BChE in fish. The activities of AST, GGT, LDH, and ALP were significantly increased in the plasma of fish exposed to diazinon. Results displayed that administration of silymarin could restore AST, GGT, LDH, and ALP activities in fish exposed to diazinon (Figure 1). Although no significant change was observed in ALT activity in fish exposed to diazinon compared with the control group on the 7th day of the experiment, its activity was significantly increased on the 14th and 21st days of the trial. Feeding fish with silymarin restored ALT activity in fish exposed to diazinon (Figure 1). LDH and CPK activities in the treated fish with diazinon were significantly higher than the control group. Moreover, administration of silymarin could not restore LDH and CPK activities in fish exposed to diazinon (Figure 1). Results indicated a significant decrease in BChE activity in fish exposed to diazinon. Although administration of silymarin improved BChE activity in fish exposed to diazinon, the activity of BChE was not restored to the normal range (Figure 1).

**Figure 2:**
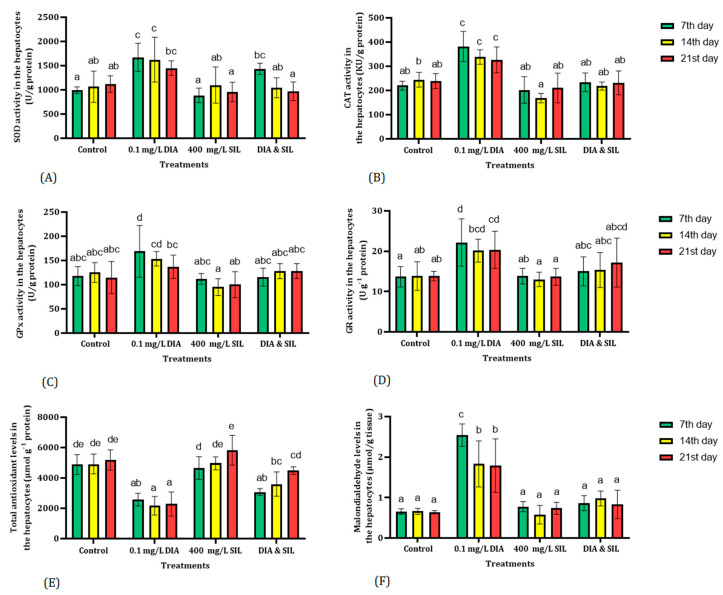
The effect of diazinon, silymarin, diazinon + silymarin on oxidative biomarkers. Values with distinct letters in a column indicate a significant difference (*p* < 0.01). Superoxide dismutase (**A**), catalase (**B**), glutathione peroxidase (**C**), glutathione reductase (**D**), total antioxidants (**E**), and malondialdehyde (**F**).

Figure 2 depicts the alterations in oxidative biomarkers in the hepatocyte of fish. The SOD activities were significantly (*p* < 0.01) increased in the plasma of fish following exposure to diazinon on the 7th and 14th days of the experiment. However, feeding fish with silymarin could restore SOD activity in fish exposed to diazinon (Figure 2). Although CAT activity was significantly (*p* < 0.01) increased in fish exposed to diazinon, administration of silymarin reduced CAT activity in treated fish with diazinon (Figure 2). The highest activity of GPx activity was observed in the hepatocytes of fish exposed to diazinon on the 7th day of the trial. However, no significant (*p* > 0.01) changes were detected in GPx activity in other treatments compared with the control group (Figure 2). Fish exposed to diazinon exhibited a notable increase in GR activity. Additionally, administering silymarin to the fish did not have any effect on adjusting GR activity in those treated with diazinon. The total antioxidant levels in the hepatocytes of fish exposed to diazinon were significantly lower than in the control group. Although feeding fish with silymarin could not restore total antioxidant capacity in fish exposed to silymarin on the 7th and 14th days of the experiment, total antioxidant levels were significantly (*p* < 0.01) increased on the 21st day (Figure 2). Exposure to diazinon led to an increase in MDA contents in the hepatocytes of fish on the 7th, 14th, and 21st days. However, no significant difference in MDA levels between the control group and fish received silymarin supplementary. Administration of silymarin could restore MDA levels to normal range in the fish exposed to diazinon. Results showed that silymarin did not have a significant effect on oxidative biomarkers in the hepatocyte of fish (Figure 2).

## 4. Discussion

Monitoring fish blood biochemical parameters and oxidative biomarkers is crucial for assessing their health. These parameters provide insights into the fish’s physiological and metabolic condition, serving as indicators for diseases, nutritional deficiencies, and environmental stressors. In ecotoxicology studies, these parameters are particularly important in order to evaluate the effects of environmental contaminants on fish well-being and metabolic functions [20,26,33].

When fish are exposed to diazinon, it can be absorbed through their gills and skin or ingested orally if it is present in the water or their food sources. Once inside the fish’s body, diazinon can affect various organs, including the liver. The increased activity of AST and ALT in the plasma of exposed fish suggested that their liver cells have been damaged, causing the release of these enzymes into the bloodstream. This increase in enzyme activity serves as a biomarker or indicator of liver injury or dysfunction. It was indicated by the results of the present study that the activity of AST and ALT in fish exposed to diazinon was regulated by the administration of silymarin, suggesting a potential beneficial effect of silymarin on mitigating liver damage caused by diazinon exposure.

Monitoring GGT activity alongside glutathione levels and other markers of oxidative stress can provide insights into the status of cellular antioxidant defenses and detoxification capacity. Elevated GGT levels in fish exposed to diazinon suggested a disturbance in liver function and could serve as an indicator of insecticide-induced hepatotoxicity [34,35]. Increasing GGT activity can accelerate the breakdown of glutathione, reducing its availability for detoxification processes and potentially compromising the cell’s ability to eliminate harmful substances [36]. As a result, reduced levels of glutathione due to increased GGT activity may lead to decreased protection against oxidative damage, increasing the susceptibility of cells to oxidative stress. The disruptions in glutathione metabolism resulting from elevated GGT activity can also interfere with redox signaling and contribute to oxidative stress. Silymarin extract has the potential to counteract the harmful effects of ROS generated by pesticides, thereby preventing or minimizing oxidative stress. This protective action can help maintain the integrity of liver cells and potentially reduce the need for elevated GGT activity. Additionally, silymarin might assist in maintaining optimal glutathione levels, which are essential for detoxification processes and the regulation of GGT. By preserving adequate glutathione levels, silymarin indirectly influences GGT activity. Moreover, silymarin might modulate gene expression, including genes involved in antioxidant defenses and liver function. This modulation has the potential to impact GGT activity and other enzymes associated with liver metabolism [37].

An increase in LDH activity indicated organ-specific toxicity and reflected the severity of cellular injury in animals exposed to diazinon. Results suggested that affected organs or tissues might be damaged or dysfunctional due to diazinon exposure. Furthermore, elevated LDH activity could also be a consequence of oxidative stress caused by diazinon, generating reactive oxygen species (ROS) and subsequent cellular damage. Therefore, monitoring LDH activity could help assess organ-specific toxicity and determine the severity of cellular injury resulting from insecticide exposure [32,38]. Results displayed that the administration of silymarin could have the potential to mitigate the increase in LDH activity caused by diazinon exposure. As LDH activity is closely associated with cellular damage and oxidative stress, administering silymarin might help stabilize LDH levels. By mitigating oxidative stress and protecting cells from damage, silymarin could have the potential to prevent or reduce the rise in LDH activity caused by exposure to diazinon.

Increased ALP activity could indicate potential liver damage or dysfunction when fish were exposed to diazinon. Elevated levels of ALP activity in the bloodstream of fish could be a sign of liver injury or cholestasis, which is the obstruction of bile flow [32,39]. Results documented the administration of silymarin has been shown to restore ALP activity and mitigate the adverse effects induced by diazinon exposure. Celep and Gedikli [40] reported the protective effect of silymarin in treating hepatotoxicity in rats.

Diazinon might disrupt cellular homeostasis and induce oxidative stress and inflammation, which could lead to tissue damage, including muscle tissue. This damage could result in the release of CPK from the damaged cells into the bloodstream, leading to increased activity [30,32]. Furthermore, diazinon might directly interfere with CPK activity by binding to the enzyme or disrupting its function. These direct interactions could impair the enzyme’s ability to catalyze the conversion of creatine phosphate, leading to an accumulation of CPK in the blood and an increase in its activity. Results showed that administration of silymarin could not restore CPK activity to normal range in fish exposed to diazinon.

BChE and acetylcholinesterase share similar structures and functions, making them susceptible to inhibition by organophosphate pesticides like diazinon. Therefore, monitoring BChE activity is a valuable biomarker used to assess exposure and toxicity to organophosphate pesticides like diazinon. A reduction in BChE activity in fish exposed to diazinon is a common indication of pesticide poisoning. Diazinon, an organophosphate pesticide, inhibits the activity of acetylcholinesterase, an enzyme responsible for breaking down acetylcholine, a neurotransmitter involved in nerve signaling [29,41]. Exposure to diazinon leads to pesticide binding to BChE, inhibiting its activity, and causing a decrease in BChE levels. This decline in BChE activity suggested that the diazinon has disrupted the normal function of the enzyme, potentially leading to the accumulation of butyrylcholine and prolonged nerve signals. Disruptions in cholinergic signaling can result in neurotoxic effects such as muscle spasms, tremors, respiratory difficulties, and paralysis. Results displayed that silymarin supplementation could not return BChE activity to the normal range in fish exposed to diazinon. Although administering silymarin to fish exposed to pesticides might help reduce oxidative stress and potentially preserve the activity of BChE, the effectiveness of silymarin in regulating BChE activity could depend on various factors, including the specific composition of antioxidants in silymarin, dosage, timing of administration, and the mechanisms by which the pesticide affects BChE activity.

Total protein in plasma refers to the concentration of all proteins present in the liquid component of blood. Changes in total protein levels can indicate various factors, such as nutritional status, liver and kidney function, inflammation, and certain diseases or conditions. The decrease in total protein levels observed after fish exposure to diazinon suggests an impact on protein metabolism. Decreased total protein levels may indicate malnutrition, liver or kidney disease, or protein-losing conditions, which can affect overall fish health [42]. On the other hand, the results showed that a silymarin supplement can increase total protein levels in fish plasma [33]. Silymarin’s antioxidant and anti-inflammatory properties may protect liver cells and promote normal functioning, leading to increased protein production in fish exposed to diazinon.

A significant decrease in albumin levels suggested that diazinon exposure could affect the liver’s ability to produce or maintain normal albumin. Reduced albumin may lead to imbalances in fluid distribution, impaired transport of essential substances, and compromised immune function. Results also displayed that exposure to diazinon could potentially lead to a decrease in globulins in the plasma of fish [38]. Diazinon might interfere with the production or metabolism of globulins, resulting in reduced levels of these proteins in the bloodstream. These changes could negatively impact the overall well-being and physiological processes of the exposed fish. Results suggested silymarin might have the potential to enhance the liver’s capacity to generate vital proteins like albumin and globulins by diminishing oxidative stress and inflammation in the liver.

The findings indicated that diazinon has the potential to interfere with the regular regulation of glucose metabolism in fish, resulting in increased levels of blood glucose. Exposure to diazinon can induce oxidative stress, which in turn disrupts the proper functioning of glucose metabolism in fish [38,43]. However, silymarin, known for its antioxidant properties, has been shown to scavenge free radicals, reduce lipid peroxidation, and enhance the activity of antioxidant enzymes. By reducing oxidative stress, silymarin may indirectly help regulate glucose levels in fish exposed to diazinon. This study revealed that silymarin exhibited specific impacts on glucose metabolism. Silymarin could have the potential to enhance insulin sensitivity, augment cellular glucose uptake, and diminish glucose production in the liver [44]. Furthermore, silymarin has hepatoprotective effects, safeguarding liver cells from toxin-induced damage. Since the liver plays a vital role in glucose metabolism, preserving liver function through silymarin supplementation could contribute to maintaining glucose homeostasis in fish exposed to diazinon.

Exposure to diazinon could potentially interfere with the regulation of cholesterol metabolism in fish. Diazinon might affect the enzymes involved in cholesterol synthesis or breakdown, leading to an accumulation of cholesterol in the fish’s tissues. Alternatively, diazinon exposure could disrupt the balance between cholesterol synthesis and excretion, resulting in elevated cholesterol levels [38,45]. Silymarin could help mitigate the toxic effects of diazinon on the liver, which is responsible for cholesterol synthesis and regulation. Results demonstrated that silymarin had the potential to restore cholesterol levels to normal concentrations in fish by safeguarding the liver from diazinon-induced damage.

When fish are exposed to diazinon, it could disrupt their lipid metabolism, leading to an increase in triglyceride levels. This disruption might occur due to the toxic effects of diazinon on the liver, which is responsible for regulating lipid metabolism in fish [38,45]. Given that silymarin has been found to support liver health and lipid metabolism in certain contexts, it is plausible that it could have a positive impact on triglyceride levels in fish exposed to diazinon. Moreover, silymarin can affect various enzymes and proteins involved in lipid metabolism. It inhibits the activity of lipogenic enzymes, which are responsible for the synthesis of triglycerides. This inhibition reduces the production of new triglycerides in the liver [46,47].

Creatinine is a waste product produced by muscle metabolism and is filtered out of the blood by the kidneys. Therefore, elevated levels of creatinine in plasma could indicate impaired kidney function. Exposure to diazinon could induce oxidative stress, inflammation, and renal tissue damage in fish. These effects could interfere with normal kidney function, potentially affecting the metabolism and excretion of urea and uric acid. Urea is a byproduct of protein metabolism, while uric acid is derived from purine metabolism [38,45]. Both substances are eliminated from the body through the kidneys, and an increase in their plasma levels could serve as indicators of renal dysfunction. Results showed the effects of silymarin were significant on kidney function and markers of kidney damage, including creatinine, urea, and uric acid. This study displayed that silymarin exhibited antioxidant and anti-inflammatory properties that could mitigate oxidative stress and inflammation within the kidneys. These beneficial effects had the potential to safeguard the integrity and functionality of kidney cells, thereby preventing the buildup of waste products in the bloodstream.

The findings suggested that exposure to diazinon led to significantly higher plasma ammonia levels in fish compared to other experimental groups. Ammonia is a toxic waste product that can accumulate in the body when the liver is unable to effectively convert it into less harmful substances. Fish excrete ammonia primarily through their gills via a process called ammonotelism. Ammonia diffuses across the gill membranes and is released into the surrounding water. Therefore, an increase in blood ammonia levels in fish exposed to diazinon displayed a disturbance in the gills’ ability to effectively excrete ammonia. Moreover, the administration of silymarin did not appear to reduce the level of ammonia in the blood of fish exposed to diazinon. Results suggested that silymarin may not have been effective in mitigating the gill damage caused by diazinon exposure or restoring normal ammonia excretion.

The increase in SOD activity indicated that the fish’s body was responding to the oxidative stress caused by diazinon exposure [2,48]. However, the results also mentioned that feeding the fish with silymarin could restore SOD activity in fish exposed to diazinon. This finding suggested that silymarin might have a protective effect against the oxidative stress induced by diazinon. It is possible that silymarin acted as an antioxidant itself or enhanced the fish’s own antioxidant defense mechanisms, including SOD activity [19].

CAT is an enzyme found in cells that helps break down hydrogen peroxide into water and oxygen, thereby protecting cells from oxidative damage. Increased CAT activity is often associated with increased oxidative stress or exposure to toxic substances. The observed increase in CAT activity in fish exposed to diazinon suggested that the fish were responding to the oxidative stress induced by the insecticide [42,48]. These findings indicated that silymarin could have modulated the oxidative stress response in the fish. By reducing CAT activity, silymarin might have helped restore the balance of antioxidant enzymes and prevented excessive ROS accumulation. The protective effect of silymarin on CAT and SOD activity is consistent with the report presented by Soto et al. [49], Soto et al. [50], and Shaarawy et al. [51] in rats exposed to alloxan and N-nitrosodiethylamine, respectively.

GPx is an important antioxidant enzyme that helps protect cells from oxidative damage by reducing hydrogen peroxide and lipid hydroperoxides. The increased GPx activity in the fish exposed to diazinon indicated a response to the oxidative stress caused by the pesticide. The hepatoprotective properties of silymarin on GPx activity were reported by Tasduq et al. [52].

The finding that GR activity was significantly increased in fish exposed to diazinon implied that the pesticide triggered oxidative stress in the fish. This could be due to the generation of ROS as a result of diazinon exposure. The increased GR activity could be seen as an adaptive response by the fish to counteract the oxidative damage caused by the pesticide. This result suggested that feeding the fish with silymarin, a natural compound with antioxidant properties, did not have an effect on adjusting GR activity in the fish treated with diazinon. Nonetheless, the protective effect of silymarin on GR and GPx activity was studied in mice exposed to different xenobiotics [53,54].

Results suggested that exposure to diazinon resulted in decreased total antioxidant levels in the hepatocytes of fish compared to a control group. Antioxidants are important molecules that help protect cells from oxidative damage caused by free radicals [55]. The results indicated that feeding fish with silymarin did not restore the total antioxidant capacity on the 7th and 14th days of the experiment. However, on the 21st day, there was a significant increase in total antioxidant levels in the fish exposed to both diazinon and silymarin compared to the control group. These findings suggested that silymarin supplementation might have a delayed restorative effect on the total antioxidant capacity in fish exposed to diazinon. It is worth noting that the increase in total antioxidant levels on the 21st day of the experiment indicated a potential protective effect of silymarin against oxidative stress induced by diazinon exposure. Kim et al. [56] found that the total antioxidant capacity in the hepatocytes of olive flounder (*Paralichthys olivaceus*) was enhanced by feeding them a diet supplemented with micelle silymarin.

Exposure to diazinon resulted in an increase in MDA contents in the hepatocytes of fish. MDA is a marker of oxidative stress and lipid peroxidation, indicating potential damage to cells [12]. Furthermore, the administration of silymarin was found to restore MDA levels to the normal range in the fish exposed to diazinon. This result indicated that silymarin had a protective effect against the oxidative damage induced by diazinon. A significant decrease in MDA contents was reported in the hepatocytes of olive flounder (*P. olivaceus*) fed with silymarin [56].

Studies have demonstrated that diazinon and its metabolites can induce oxidative stress and damage cell membranes through lipid peroxidation [57]. This occurs when reactive oxygen species (ROS) generated by diazinon exposure react with polyunsaturated fatty acids in the cell membrane, leading to the formation of lipid peroxides. Lipid peroxidation can disrupt the fluidity and permeability of the cell membrane, affecting its structural integrity and function [13].

Silymarin is a mixture of flavonolignans extracted from the milk thistle plant (*S. marianum*). It is known for its antioxidant properties and has been studied for its potential protective effects against various toxins and oxidative stress-related conditions [58]. In this case, silymarin seems to have counteracted the oxidative stress induced by diazinon exposure in fish, possibly by scavenging reactive oxygen species (ROS) or enhancing the fish’s antioxidant defense system. Studies showed that silymarin can directly interact with ROS molecules, donating an electron to stabilize them and prevent them from causing damage [59]. Furthermore, silymarin can stimulate the production of endogenous antioxidants, such as glutathione, which help to counteract the harmful effects of ROS [60]. Also, silymarin has the ability to bind to metal ions, such as iron and copper, which can catalyze the formation of ROS. By chelating these metals, silymarin helps to inhibit ROS production [61,62].

Studies showed that most pesticides could damage DNA, cause apoptosis, oxidize proteins and lipids in non-target organisms by disrupting mitochondrial Ca^2+^ levels, respiration, and triggering ROS production [63,64,65,66].

Silymarin contains several active compounds, primarily silybin, silydianin, and silychristin, which exhibit antioxidant properties [67]. Martínez et al. [68] found that sylibin, as the active ingredient of silymarin, could exert its protective effect on cells exposed to pesticides by regulating the expression of genes involved in inflammation, apoptosis and oxidative stress. Research on silymarin suggests that it can protect against oxidative stress by increasing the activity of antioxidant enzymes such as SOD, CAT, and GPx. Silymarin can also reduce the production of ROS and inhibit lipid peroxidation, which is the oxidative degradation of lipids in cell membranes [69]. Moreover, silymarin can enhance the levels of glutathione (GSH), an important endogenous antioxidant in the body [69].

## 5. Conclusions

In conclusion, exposure to diazinon can harm fish organs and processes. Enzyme levels, like AST, ALT, GGT, LDH, and ALP, indicate liver damage due to diazinon exposure. Silymarin may help restore enzyme activity but might not affect CPK or BChE. Diazinon disrupts protein metabolism, reducing total protein, albumin, and globulin levels. Silymarin can increase these levels in exposed fish. Diazinon affects glucose and lipid metabolism, but silymarin’s impact on triglycerides is uncertain. Diazinon exposure can harm kidneys, while silymarin protects them. Gill damage and ammonia excretion are not improved by silymarin. Silymarin shows potential in mitigating liver damage and improving fish health, but more research is needed for a full understanding. Furthermore, the results of this study suggested that silymarin supplementation might have a protective effect against the oxidative stress induced by diazinon exposure in fish. The increase in SOD activity and restoration of SOD activity by silymarin indicated its potential to enhance the fish’s antioxidant defense mechanisms. Additionally, silymarin may have modulated the oxidative stress response by reducing CAT activity and restoring the balance of antioxidant enzymes. Although silymarin did not immediately restore the total antioxidant capacity, it showed a delayed restorative effect on the 21st day of the experiment. Furthermore, silymarin was found to restore MDA levels to the normal range, indicating its ability to protect against oxidative damage and lipid peroxidation. Overall, these findings highlight the potential of silymarin as a natural compound with antioxidant properties to mitigate the adverse effects of diazinon-induced oxidative stress in fish. However, it is important to note that while these findings are encouraging, there is still limited research specifically focusing on the effects of silymarin against pesticide-induced oxidative stress. Further studies are necessary to determine its effectiveness and the appropriate dosage for this particular purpose. Furthermore, extra research is warranted to explore the underlying mechanisms and evaluate the efficacy of silymarin in different experimental settings.

## Figures and Tables

**Table 1:** The effect of diazinon, silymarin, diazinon + silymarin on the blood biochemical parameters.

Day	Treatments	Biochemical Parameters					
		Total Protein (g dL^−1^)	Albumin (g dL^−1^)	Globulins (g dL^−1^)	Glucose (mg dL^−1^)	Triglycerides (mg dL^−1^)	Cholesterol (mg dL^−1^)
7th day	Control	4.9 ± 0.6 ^c^	2.4 ± 0.4 ^b^	2.5 ± 0.4 ^c^	61.9 ± 3.4 ^bc^	284.8 ± 31.3 ^ab^	238.0 ± 26.3 ^abc^
	0.1 mg L^−1^ DIZ	3.6 ± 0.3 ^a^	1.8 ± 0.2 ^a^	1.8 ± 0.2 ^a^	73.1 ± 5.7 ^d^	336.7 ± 28.0 ^b^	324.2 ± 59.1 ^b^
	400 mg kg^−1^ SIL	4.8 ± 0.6 ^c^	2.3 ± 0.3 ^ab^	2.4 ± 0.3 ^c^	61.3 ± 4.8 ^bc^	246.1 ± 33.4 ^a^	224.6 ± 45.4 ^ab^
	SIL + DIZ	4.5 ± 0.3 ^bc^	2.2 ± 0.4 ^ab^	2.3 ± 0.4 ^bc^	64.9 ± 2.3 ^c^	288.8 ± 33.2 ^ab^	223.3 ± 22.5 ^ab^
14th day	Control	4.5 ± 0.6 ^bc^	2.2 ± 0.4 ^ab^	2.3 ± 0.4 ^c^	61.7 ± 4.9 ^bc^	318.1 ± 44.2 ^b^	254.7 ± 32.7 ^bc^
	0.1 mg L^−1^ DIZ	3.7 ± 0.3 ^a^	1.9 ± 0.2 ^ab^	1.9 ± 0.2 ^ab^	76.8 ± 2.8 ^d^	293.6 ± 32.8 ^ab^	281.8 ± 21.6 ^cde^
	400 mg kg^−1^ SIL	4.9 ± 0.9 ^c^	2.3 ± 0.4 ^b^	2.5 ± 0.5 ^c^	57.9 ± 8.2 ^ab^	243.9 ± 37.4 ^a^	215.7 ± 41.8 ^ab^
	SIL + DIZ	4.1 ± 0.3 ^ab^	1.9 ± 0.1 ^a^	2.2 ± 0.3 ^abc^	67.0 ± 3.4 ^c^	259.3 ± 22.7 ^a^	247.4 ± 36.5 ^abc^
21st day	Control	4.7 ± 0.7 ^bc^	2.3 ± 0.3 ^b^	2.4 ± 0.4 ^c^	61.6 ± 4.3 ^bc^	290.0 ± 52.6 ^ab^	267.3 ± 38.0 ^bcd^
	0.1 mg L^−1^ DIZ	3.7 ± 0.5 ^a^	2.0 ± 0.3 ^ab^	1.8 ± 0.2 ^a^	73.6 ± 2.9 ^d^	327.1 ± 36.3 ^b^	318.7 ± 39.7 ^de^
	400 mg kg^−1^ SIL	4.9 ± 0.4 ^c^	2.2 ± 0.3 ^ab^	2.7 ± 0.6 ^c^	55.1 ± 3.1 ^a^	244.0 ± 42.4 ^a^	198.1 ± 37.9 ^a^
	SIL + DIZ	4.6 ± 0.3 ^bc^	2.1 ± 0.3 ^ab^	2.5 ± 0.4 ^c^	63.3 ± 4.4 ^bc^	286.0 ± 42.3 ^ab^	267.0 ± 52.7 ^bcd^

Values with distinct letters in a column indicate a significant difference (*p* < 0.01).

**Table 2:** The effect of diazinon, silymarin, diazinon + silymarin on plasma metabolites.

Day	Treatments	Plasma Metabolites			
		Creatinine (mg dL^−1^)	Urea (mg dL^−1^)	Uric Acid (mg dL^−1^)	Ammonia (mg dL^−1^)
7th day	Control	0.28 ± 0.11 ^a^	0.91 ± 0.03 ^a^	0.23 ± 0.04 ^ab^	0.63 ± 0.12 ^a^
	0.1 mg L^−1^ DIZ	0.58 ± 0.12 ^b^	1.61 ± 0.21 ^b^	0.30 ± 0.06 ^c^	1.98 ± 0.37 ^d^
	400 mg kg^−1^ SIL	0.37 ± 0.07 ^a^	0.88 ± 0.13 ^a^	0.20 ± 0.02 ^a^	0.67 ± 0.10 ^a^
	SIL + DIZ	0.31 ± 0.08 ^a^	0.94 ± 0.17 ^a^	0.27 ± 0.07 ^abc^	1.74 ± 0.48 ^cd^
14th day	Control	0.36 ± 0.12 ^a^	0.91 ± 0.15 ^a^	0.21 ± 0.04 ^a^	0.72 ± 0.26 ^a^
	0.1 mg L^−1^ DIZ	0.91 ± 0.18 ^c^	1.57 ± 0.27 ^b^	0.32 ± 0.05 ^c^	1.74 ± 0.38 ^cd^
	400 mg kg^−1^ SIL	0.32 ± 0.11 ^a^	0.89 ± 0.12 ^a^	0.26 ± 0.03 ^abc^	0.50 ± 0.08 ^a^
	SIL + DIZ	0.26 ± 0.14 ^a^	0.91 ± 0.12 ^a^	0.26 ± 0.09 ^abc^	1.67 ± 0.31 ^bcd^
21st day	Control	0.29 ± 0.14 ^a^	0.89 ± 0.16 ^a^	0.21 ± 0.03 ^a^	0.69 ± 0.12 ^a^
	0.1 mg L^−1^ DIZ	0.86 ± 0.20 ^c^	1.69 ± 0.32 ^b^	0.30 ± 0.04 ^c^	1.49 ± 0.37 ^bc^
	400 mg kg^−1^ SIL	0.31 ± 0.12 ^a^	0.88 ± 0.23 ^a^	0.21 ± 0.06 ^a^	0.66 ± 0.20 ^a^
	SIL + DIZ	0.32 ± 0.11 ^a^	0.99 ± 0.23 ^a^	0.28 ± 0.05 ^bc^	1.32 ± 0.44 ^b^

Values with distinct letters in a column indicate a significant difference (*p* < 0.01).

## Data Availability

All data that found in this study were presented as Tables and Figures.
